# Sem fôlego: os aprendizados para derrotar nosso próximo vírus mortal

**DOI:** 10.1590/0102-311XPT218024

**Published:** 2025-02-24

**Authors:** Elissandro Fonseca dos Banhos, Ariela Soraya do Nascimento Siqueira

**Affiliations:** 1 Universidade Federal do Oeste do Pará, Santarém, Brasil.

O mundo se impressiona quando uma criatura com poucos micrômetros de tamanho ameaça a vida humana de forma global. O escritor, divulgador científico e jornalista David Quammen, com certeza, não é uma dessas pessoas. Um de seus *best-sellers*, publicado em 2012 (*Spillover: Animal Infections and the Next Human Pandemic* ^1^), já descrevia a forma de pensar desse autor sobre esse tema.

O livro ora resenhado (*Sem Fôlego: A Corrida Ciêntífica para Derrotar um Vírus Mortal* ^2^) possui relevância acadêmico-científica por dois aspectos. Primeiro, porque o autor descreve a história do SARS-CoV-2, suas modificações e desdobramentos, de forma a analisarmos como esse tipo de ameaça biológica está relacionado com a maneira como a humanidade vem interferindo na natureza. Segundo, porque a obra ilustra o papel da pesquisa científica na proposição de estratégias para mitigar as possíveis ameaças biológicas que possam surgir com o tempo.

Os oito capítulos em que o livro está dividido são: I - *Os Cidadãos não Precisam Entrar em Pânico*; II - *Os Avisos*; III - *Mensagem numa Garrafa*; IV - *Dinâmica de Mercado*; V - *Variáveis e Constantes*; VI - *Quatro Tipos de Magia*; VII - *Os Leopardos de Mumbai*; VIII - *Ninguém Sabe Tudo*. Capítulos escritos a partir de entrevistas com cerca de 95 pessoas, entre pesquisadores e autoridades relacionadas a SARS-CoV-2 e a pandemia, que contribuíram de forma direta ou indireta com as informações para a elaboração e a produção da obra.

O primeiro capítulo, *Os Cidadãos não Precisam Entrar em Pânico*, trata da descoberta da doença, em que as autoridades e as instituições políticas precisaram tomar medidas para contenção da disseminação do vírus. O governo chinês decidiu unilateralmente que não era necessário divulgar as informações sobre a doença no país. A Organização Mundial da Saúde (OMS) se posicionou, afirmando que a pesquisa científica tem a obrigação ética de divulgar resultados, sejam eles positivos, inconclusivos ou negativos ^3^.

O segundo capítulo do livro, *Os Avisos*, enfatiza os fatos científicos que poderiam ter resultado em respostas mais rápidas em todo o mundo no combate ao vírus. Talvez o exemplo mais expressivo de que não estávamos atentos aos avisos desse tipo de microrganismo seja o surto de SARS-CoV-2 ocorrido na China em 2002 e 2003 ^4^. Até então, acreditava-se que o coronavírus causava apenas infecções respiratórias leves. Esse surto deveria ter aberto os olhos do mundo para o fato de que ele também pode causar sintomas respiratórios graves.

A *Mensagem numa Garrafa*, título do terceiro capítulo, ilustra o fato de que as informações científicas sobre o coronavírus, sobre seus reservatórios e sobre o desenvolvimento da doença vêm de forma lenta. Nele, o autor mostra como o conhecimento científico sério necessita de tempo para ser construído, para se consolidar e para se tornar útil para população. Um dos exemplos que podemos citar ocorreu em plena pandemia com a descoberta da redução do risco de contágio com o uso de máscara ^5^.

O quarto capítulo do livro marca uma mudança no foco do autor, passando a discutir como outros países lidaram com as questões envolvendo a pandemia, por isso o título *Dinâmica de Mercado*. Apesar dos avanços científicos relacionados ao vírus, inclusive com relação ao desenvolvimento de vacinas, o que parecia um avanço que beneficiaria o mundo inteiro foi transformado em um tema de discussão política, com claro viés econômico, o que prejudicou o avanço na vacinação pelo mundo ^6^.

O capítulo cinco, intitulado *Variáveis e Constantes*, buscou discutir sobre os fatores biológicos relacionados as alterações genéticas sofridas pelo vírus. Tornou-se essencial entender quais são seus mecanismos de regulação, e quais os fatores que fazem variar ou tornar constante essas características. Um exemplo da utilidade dessa informação foi a decisão tomada por parte do primeiro-ministro da Inglaterra, que aumentou a rigidez do *lockdown* em todo o país, após a divulgação de trabalhos científicos da área que mostraram que o novo vírus havia adquirido maior capacidade de transmissão.

No capítulo seis, o autor trata de temas relacionados a pandemia que, segundo ele, se assemelham a esperanças mágicas, por isso o nome *Quatro Tipos de Magia*. Quammen diz que existiram quatro tipos de esperanças mágicas relacionadas a pandemia, e nesse capítulo, discute e desconstrói conceitos equivocados, anticientíficos com os quais ele mesmo teve de lidar ao se deparar com vários discursos nessa direção.

É inevitável para o leitor da obra associar o que o autor chama de tipos de magia com o conjunto de notícias falsas, as denominadas *fake news* que tiveram enorme destaque político não apenas nos Estados Unidos, na pessoa do seu presidente à época, mas em diversos países em que esses tipos de declarações saíram do escopo do jornalismo e foram usadas como tática de marketing eleitoral ^7^.

No sétimo capítulo do livro, *Os Leopardos de Mumbai*, o autor aborda as possíveis origens do vírus, os lugares remotos e os seus mais diferentes reservatórios. Um dos reservatórios de maior relevância é o morcego. Essa ameaça se estabelece a partir do momento em que as ações humanas destroem a biodiversidade, afetando o equilíbrio na vida silvestre, que resulta em um necessário deslocamento do animal para outros ambientes, levando consigo o vírus e promovendo a dispersão viral ^8^. Esse tipo de informação deveria mover a sociedade mundial em uma direção diferente, em que os ambientes naturais fossem respeitados e conservados a sua maneira. Se não por uma consciência ambiental ou por uma valorização da biodiversidade, talvez por um receio justificado de que todo esse processo de disseminação viral aconteça novamente.

No oitavo capítulo, *Ninguém Sabe Tudo*, o autor nos lembra que o conhecimento científico tem uma característica fragmentária e provisória, predicados que são fundamentais para sua contínua modificação e avanço. Um exemplo citato no livro é o da feira de Huanan (China), ambiente caracterizado pela sua total falta de higiene e propício para uma disseminação viral, que por isso também é apontado como o local de origem da pandemia. Então, o que se espera é que, de posse desse conhecimento, possamos tomar medidas ambientais, sociais e políticas para que situações como essas da feira não se repitam e não nos coloque novamente em uma situação de ameaça a saúde.

Levando em consideração o tema da obra e a sua abordagem, podemos considerá-la como de significativa relevância para as áreas da Saúde e das Ciências Biológicas, e também para as Ciências de uma forma geral, pois utilizou o tema da COVID-19 para abordar conteúdos mais amplos sobre o desenvolvimento do método científico. Assim, deixamos nossa enfática indicação para a leitura dela aos interessados na temática das epidemias, com um olhar para o futuro. Em última análise, a obra pode contribuir com a formação de um profissional que possui melhor capacidade de, olhando para o passado, enfrentar os desafios do futuro da melhor maneira.
